# A New Mouse Model of Giant Axonal Neuropathy with Overt Phenotypes and Neurodegeneration Driven by Neurofilament Disorganization

**DOI:** 10.1523/JNEUROSCI.1959-22.2023

**Published:** 2023-05-31

**Authors:** Banshi Nath, Jean-Pierre Julien

**Affiliations:** ^1^CERVO Brain Research Centre, Québec City, Québec G1E1T2 Canada; ^2^Department of Psychiatry and Neuroscience, Université Laval, Québec City, Québec G1E1T2 Canada

**Keywords:** giant axonal neuropathy, gigaxonin, intermediate filament, neurofilament, peripherin.

## Abstract

Research on pathogenic mechanisms underlying giant axonal neuropathy (GAN), a disease caused by a deficiency of gigaxonin, has been hindered by the lack of appropriate animal models exhibiting substantial symptoms and large neurofilament (NF) swellings, a hallmark of the human disease. It is well established that intermediate filament (IF) proteins are substrates for gigaxonin-mediated degradation. However, it has remained unknown to what extent NF accumulations contribute to GAN pathogenesis. Here, we report the generation of a new mouse model of GAN that is based on crossing transgenic mice overexpressing peripherin (Prph) with mice knockout for *Gan*. The Gan^−/−^;TgPer mice developed early onset sensory-motor deficits along with IF accumulations made up of NF proteins and of Prph, causing swelling of spinal neurons at a young age. Abundant inclusion bodies composed of disorganized IFs were also detected in the brain of Gan^−/−^;TgPer mice. At 12 months of age, the Gan^−/−^;TgPer mice exhibited cognitive deficits as well as severe sensory and motor defects. The disease was associated with neuroinflammation and substantial loss of cortical neurons and spinal neurons. Giant axons (≥160 μm^2^) enlarged by disorganized IFs, a hallmark of GAN disease, were also detected in dorsal and ventral roots of the Gan^−/−^;TgPer mice. These results, obtained with both sexes, support the view that the disorganization of IFs can drive some neurodegenerative changes caused by gigaxonin deficiency. This new mouse model should be useful to investigate the pathogenic changes associated with GAN disease and for drug testing.

**SIGNIFICANCE STATEMENT** Research on pathogenic mechanism and treatment of GAN has been hampered by the lack of animal models exhibiting overt phenotypes and substantial neurofilament disorganization, a hallmark of the disease. Moreover, it remains unknown whether neurologic defects associated with gigaxonin deficiency in GAN are because of neurofilament disorganization as gigaxonin may also act on other protein substrates to mediate their degradation. This study reports the generation of a new mouse model of GAN based on overexpression of Prph in the context of targeted disruption of gigaxonin gene. The results support the view that neurofilament disorganization may contribute to neurodegenerative changes in GAN disease. The Gan^−/−^;TgPer mice provide a unique animal model of GAN for drug testing.

## Introduction

Neurofilaments (NFs) are made from the assembly of intermediate filament (IF) proteins including type IV IF proteins (Nefh, Nefm, Nefl, and α-internexin) and a type III IF protein called peripherin (Prph). NFs are involved mainly in the radial growth and structural maintenance of axons (Gafson et al., 2020). Abnormal accumulation of NFs occurs in several neurodegenerative diseases including Charcot-Marie-Tooth disease (Adebola et al., 2015), Alzheimer's disease (Ishii et al, 1979), amyotrophic lateral sclerosis (Gros-Louis et al., 2004), and giant axonal neuropathy (GAN; Berg et al., 1972; Sandelius et al., 2018). NF disorganization may also result from mutations in NF genes (Mersiyanova et al., 2000; Gros-Louis et al., 2004; Adebola et al., 2015) and the kinesin gene (Xia et al., 2003).

GAN is an autosomal recessive neurodegenerative disease characterized by the deterioration of the central and peripheral nervous system (Asbury et al., 1972; Berg et al., 1972). The onset of GAN disease phenotypes is characterized by sensory and motor track deficits that further progress into areflexia, loss of deep and superficial sensitivity, ambulatory disability, and death during the late second decade of life (Asbury et al., 1972; Bomont et al., 2003; Demir et al., 2005; Hentati et al., 2013). Sural nerve biopsies of human GAN patients have shown the presence of giant axons filled with IFs, a characteristic feature of GAN disease (Asbury et al., 1972). Mutations in the gigaxonin gene were identified as the cause of GAN (Bomont et al., 2000). Gigaxonin is a member of the bric-a-brac, tram track, broad complex (BTB)/Kelch family protein, which interacts with Cul3/ubiquitin ligase enzyme with its N-terminal BTB domain, whereas at C-terminal Kelch domain it interacts with its substrate such as IF proteins (Mahammad et al., 2013; Johnson-Kerner et al., 2015), microtubule-associated protein (MAP; Allen et al., 2005) and Sonic hedgehog (Shh) protein (Arribat et al., 2019). Gigaxonin plays a key role in maintaining the turnover of IF proteins by recruiting them toward Cul3 ubiquitin to undergo proteasomal-mediated degradation (Mahammad et al., 2013). An *in vitro* study revealed that overexpression of gigaxonin in patient-derived fibroblasts can diminish the levels of vimentin (Mahammad et al., 2013). Another study reported that gigaxonin maintains NF protein levels in iPSC-derived cultured motor neurons (Diaz et al., 2015). Since its discovery, >40 different mutations have been reported in the gigaxonin gene including missense, nonsense, frameshift, and deletion mutations (Johnson-Kerner et al., 2014). Those mutations cause a deficiency in gigaxonin levels that provokes GAN disease.

To date, three mouse models of GAN have been generated by the targeted deletion of gigaxonin sequences. The targeted deletion of Gan exons 3–5 led to accumulations of MAP proteins and NF proteins in the brain and spinal cord at the age of 24 months (Valle et al., 2006; Ganay et al., 2011). Another mouse with targeted deletion of exon 1 exhibited some NF aggregates in the nervous system but with only modest behavioral phenotypes (Dequen et al., 2008). All three mouse lines with targeted deletion of Gan exons lacked overt GAN phenotypes and neuronal loss during aging. So these Gan knock-out mice are of limited use for investigating the GAN pathology and for drug testing.

Here, we report the generation of a new mouse model of GAN which is based on crossing mice (PerTg) overexpressing a Prph transgene (Beaulieu et al., 1999) with mice lacking Gan exon 1 (Dequen et al., 2008). The PerTg mice described previously exhibited Prph accumulations and motor phenotypes, but only at a very old age (24 months; Beaulieu et al., 1999). However, when crossed with Gan knock-out mice to generate PerTg mice lacking gigaxonin, the progeny Gan^−/−^;TgPer mice developed sensory motor deficits at a young age along with large IF accumulations made up of NF proteins and Prph in the spinal cord. At 12 months of age, the Gan^−/−^;TgPer mice exhibited severe cognitive, sensory, and motor deficits with significant neuronal loss and muscle denervation. Giant axons, a hallmark of GAN disease, were also detected in dorsal and ventral roots of the Gan^−/−^;TgPer mice. This new mouse model of GAN, based on excess Prph expression in the context of gigaxonin deficiency, supports the view that IF disorganization can contribute to neurodegenerative changes in GAN disease.

## Materials and Methods

### Generation of transgenic mice

**Figure 1.**
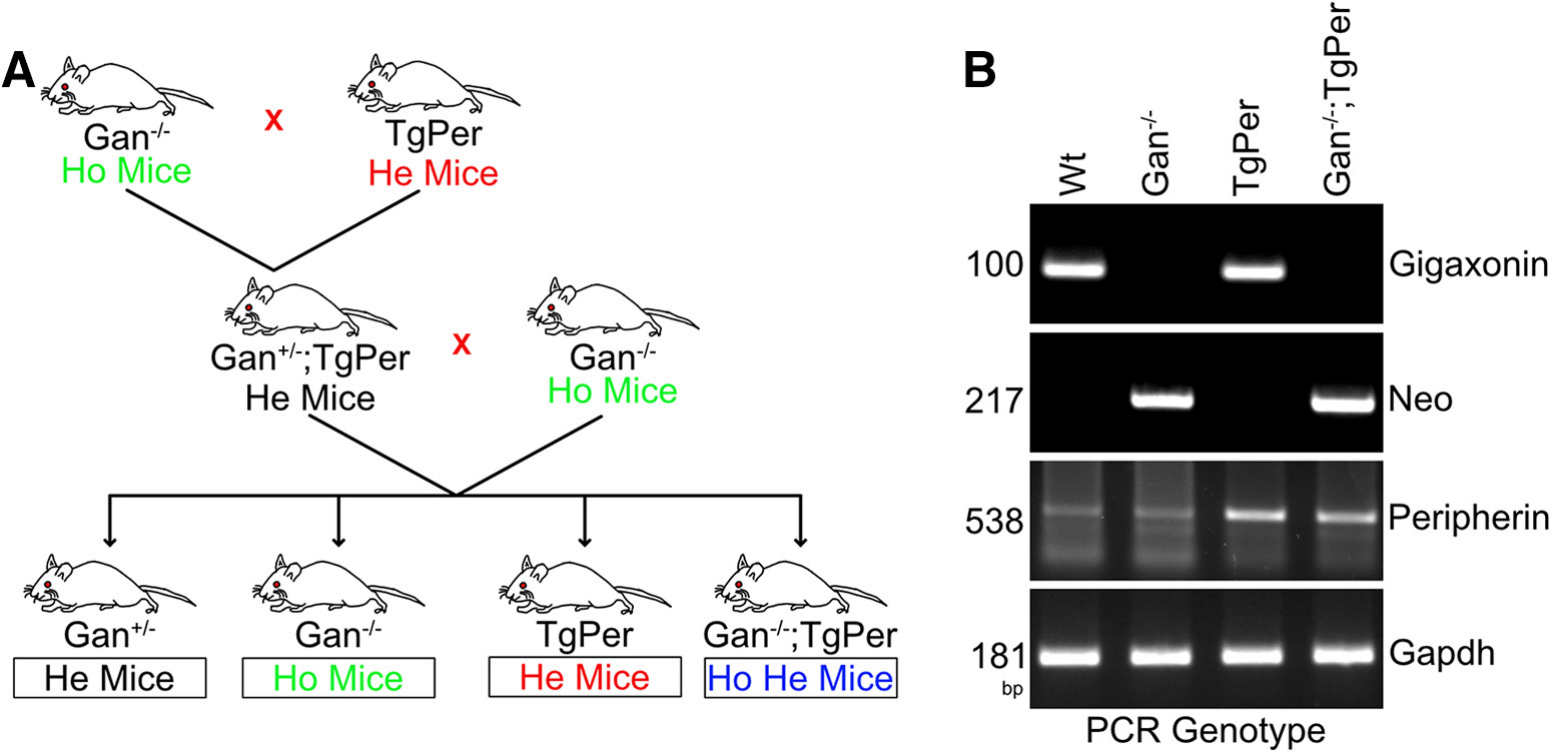
Schematic representation for the generation of Gan^−/−^;TgPer mice. ***A***, Gan^+/−^;TgPer mice were generated by breeding homozygous (Ho) Gan^−/−^ mice with heterozygous (He) TgPer mice. Then the Gan^−/−^;TgPer mice were crossed with Gan^−/−^ to generate the Gan^−/−^;TgPer mice along with Gan^−/−^ and TgPer mice. ***B***, PCR of tail DNA was used to determine the mouse genotypes for the targeted disruption of Gan gene and insertion of multiple Prph transgenes.

Gan^−/−^ mice and TgPer transgenic mice were described previously (Beaulieu et al., 1999; Dequen et al., 2008). The Gan^−/−^ mice were generated as described previously (Dequen et al., 2008). Briefly, a 1 kb sequence containing part of the gigaxonin gene promoter and translation initiation site in exon 1 was replaced by a 1.5 kb fragment of targeting vector containing a neomycin (Neo) cassette. Further, this targeting fragment was electroporated into the embryonic stem cells, which were then microinjected into blastocysts to generate chimeric mouse founders. Male chimeras were then mated with C57BL/6 background females to generate mice heterozygous for Gan exon 1 deletion. The Gan^−/−^ mice were derived from the breeding of heterozygous Gan^+/−^ mice. On the other hand, the transgenic mice overexpressing the wild-type peripherin gene (TgPer mice) were produced by microinjection into one-cell embryos of a 9.5 kb DNA fragment of an intact peripherin gene including its promoter elements, introns, and 3′ region. (Beaulieu et al., 1999). The mouse genotypes were confirmed by polymerase chain reaction (PCR) of tail DNA with the use of appropriate primers (Table 1). After confirming the genotypes of Gan^−/−^ and TgPer, these animals were used to produce the double transgenic Gan^−/−^;TgPer mice. As shown in Figure 1, the Gan^−/−^ mice were mated with TgPer transgenic mice to produce Gan^+/−^;TgPer mice, which were then crossed with Gan^−/−^ mice to produce a cohort of Gan^−/−^;TgPer mice. A semiquantitative genomic DNA PCR was conducted to confirm the Gan, Neo, glyceraldehyde 3-phosphate dehydrogenase (Gapdh) and Prph genes genes using primers for PCR of tail DNA at every step (Fig. 1*B*; Table 1). All experiments were approved by the Laval University Animal Care Ethics Committee (protocol #2020-568) and were in accordance with the *Guide to the Care and Use of Experimental Animals* of the Canadian Council on Animal Care.

**Table 1.** List of primers used for genomic tail DNA PCR

Gene	Primer	Size
Gigaxonin	Forward 5′-GTGTCCGACCCTCAGCAC-3′ Reverse 5′-GCCAGGATGTTCTTCTGCAC-3′	100 bp
Neo cassette	Forward 5′-CTTGGGTGGAGAGGCTATTC-3′ Reverse 5′-AGGTGAGATGACAGGAGATC-3′	217 bp
Peripherin	Forward 5′- ATGAGCCATCATCACTCGGGCC-3′ Reverse 5′-TCTGCTTGAGCGCCGCTAGGTCCT-3′	538 bp
GAPDH	Forward 5′-AGTGTTTCCTCGTCCCGTAG-3′ Reverse 5′-GCCGTGAGTGGAGTCATACT-3′	181 bp

### Behavior analysis and motor performance test

A novel object test was performed to assess memory impairment in mice. On day 1, mice from both sexes were allowed to habituate themselves freely to the experimental cage (Plexiglas box) for 180 s. On day 2, two similar (same shape, color, and odorless) objects were placed on predetermined spots on the floor. Mice were allowed to explore the objects for the same duration. On day 3, one of the objects (different shape, color, and odorless) was replaced with a familiar object, and mice were again freely allowed to explore. Exploratory time spent by the mice on both familiar and novel objects was recorded for the same duration of time by a person blinded to the experiment. The total percentage of time spent on either novel or familiar objects was calculated. To assess the anxiety in mice, we performed an open field test using a VersaMax system (Omnitech Electronics.). Mice were placed in the middle of the VersaMax platform and allowed to walk freely in the dark for 5 × 6 min. The total percentage of time spent in the center was calculated to check anxiety, and the total distance traveled in the periphery was analyzed to check the locomotor impairment. To assess the sensory deficits, the hot plate test was performed at temperatures of 50°C and 52°C. Briefly, the mice were placed on the hot plate, and latency to retract the paw (in seconds) from the plate was recorded. The cutoff time for the hot plate was set for 40 s. Hindlimb extension, rotarod, grid, and footprint tests were performed to assess motor impairment. To evaluate the motor performance, mice were trained to run on an accelerating rotarod machine at a speed of 3 rpm with 0.1 rpm/acceleration. All four mice groups (WT, Gan^−/−^, TgPer, and Gan^−/−^;TgPer) were trained for 3 d before taking the final recording. Cutoff time was set at 300 s for all mice, and the longest latency to fall from the rotating rod was used for analysis. Hindlimb strength for the four groups was assessed by the grid hang test. Mice were placed on a grill that was inverted gently. The cutoff time for the grill hang test was 90 s. Hindlimb extension was checked by gently holding the mice in the air for 10 s, and the capacity of the hindlimb extension of the mice was recorded. The extension rating was set at up to three, with zero being complete retraction and three being fully extended hindlimb. A mice footprint test was performed to check gait impairment and motor loss. A homemade test corridor was constructed using a 90 × 8 cm ramp. Forelimb and hindlimb paws were painted green and red, and the mice were allowed to walk freely on a paper on the ramp. The stride length of both forelimbs and hindlimbs was measured from the middle of the footprint.

### Tissue collection and sample preparation for microscopy

Mice from both sexes were anesthetized by intraperitoneal injection of ketamine (10 mg/ml) and xylazine (1 mg/ml) before being killed. Transcardial perfusion was performed with ice-cold PBS followed by 4% PFA. Brain, spinal cord, and muscles were collected and stored in 4% PFA overnight followed by 30% sucrose storage at 4°C, whereas dorsal root ganglia (DRG) were stored in 1% glutaraldehyde. Transverse sections 25 µm thick were cut using a sliding VT1200 S microtome (Leica). For immunostaining, sections were mounted on a glass slide and allowed to dry overnight. Sections were washed 3× with PBS followed by an antigen retrieval method using 10 mm sodium citrate, 5% Tween 20, pH 6, at boiling temperature for 20 min. Slides were washed with PBS 2× at room temperature followed by 3× washing with 0.25% Triton X-100 in PBS (PBST). Blocking was performed with 10% goat serum (Invitrogen) in PBST for 2 h to reduce nonspecific antibody binding. After blocking, sections were incubated with suitable primary antibody (Table 2) overnight at 4°C. The next day, slides were washed 3× with PBST for 5 min each followed by appropriate secondary Alexa Fluor antibody (Table 2) in PBS for 2 h at room temperature in a dark chamber. After washing, slides were briefly incubated in DAPI (1:10 000; Invitrogen) for 1 min followed by 1× TrueBlack Lipofuscin Autofluorescence Quencher (Biotium) incubation for 1 min prepared in 70% ethanol. After several washes of PBS, slides were mounted with Fluoromount-G (SouthernBiotech). The images were acquired from the cerebral cortex area in the brain and from the ventral horn area of the lumbar region (L4/L5) of the spinal cord for analysis using Nikon A1 confocal microscope, 25× or 63× oil immersion objective. To assess the neurodegeneration, a Nissl stain was used. Briefly, brain and spinal cord sections were washed in Milli-Q followed by serial alcohol dehydration (50, 70, 90, and 100%) and 2× xylene for 3 min. Sections were incubated in 1% Cresyl violet stain for 1 min, followed by rehydration in xylene and alcohol for 3 min each, mounted with DPX mounting media (Sigma-Aldrich), and visualized under a light field microscope (Leica DM5000 B microscope) at 20×.

**Table 2.** Details of antibodies used for immunofluorescence and immunoblotting

Antibody	Species	Clone	Company	Catalog no.	Western	IHC	Temperature	Time
Anti-NFH (SMI32)	Mouse mono		BioLegend	801701	1:1000	1:1000	4°C	ON
Anti- NFM	Mouse mono	NN18	Sigma-Aldrich	N5264	1:1000	1:500	4°C	ON
Anti-NFL	Mouse mono	NR4	Sigma-Aldrich	N5139	1:1000	1:500	4°C	ON
Anti-α-internexin	Mouse mono	2E3	Sigma-Aldrich	I0283	1:1000	1:500	4°C	ON
Anti-peripherin	Rabbit poly		Abcam	Ab4666	1:1000	1:1000	4°C	ON
Anti-Actin	Rabbit mono	13E5	Cell Signalling Technology	mAb4970	1:5000		4°C	ON
Anti-Iba1	Rabbit		Wako Chemicals	019-19741	1:1000	1:1000	4°C	ON
Anti-GFAP	Mouse mono		Cell Signalling Technology	3670	1:1000	1:1000	4°C	ON
Anti- Vimentin	Mouse mono		Millipore	IF01	1:1000		4°C	ON
Anti-NeuN	Mouse mono	A60	Millipore	MAB377		1:1000	4°C	ON
Anti-NeuN	Rabbit mono	D3s31	Cell Signalling Technology	12943		1:1000	4°C	ON
Anti-TDP-43	Rabbit poly		Proteintech	10782-2-AP		1:500	4°C	ON
Anti-TDP-43	Mouse mono	E10	Santa Cruz Biotechnology	SC-376311		1:250	4°C	ON
Anti-α-Tubulin	Rabbit poly		Cell Signalling Technology	2144	1:1000		4°C	ON
Anti-Mouse IgG-HRP (H + L)	Goat poly		Jackson ImmunoResearch	115-035-144	1:5000		RT	1 h
Anti-Rabbit IgG-HRP (H + L)	Goat poly		Jackson ImmunoResearch	111-035-003	1:5000		RT	1 h
Alexa Fluor 488 Anti-Mouse IgG (H + L)	Goat poly		Thermo Fisher Scientific	A-11029		1:500	RT	2 h
Alexa Fluor 594 Anti-Rabbit IgG (H + L)	Goat poly		Thermo Fisher Scientific	A-11012		1:500	RT	2 h

### Western blotting

Brains and spinal cords were collected as described above. Briefly, mice were anesthetized by the method previously described. Transcardial blood perfusion was performed using ice-cold PBS, followed by tissue collection, and stored at −80°C. Tissue was homogenized immediately after collection to separate the soluble and insoluble fractions as previously reported (Dutta et al., 2020). Briefly, the brain and spinal cord samples were homogenized in freshly prepared RIPA buffer (50 mm Tris, pH 7.4, 1 mm EDTA, 150 mm NaCl, 0.25% SDS, 1% NP-40, 0.1 mm DTT, 1× protease inhibitor, and 1× phosphatase inhibitor cocktail) followed by 30 min homogenization at 4°C and centrifuged at 12,000 × *g* for 20 min at 4°C. After centrifugation, the supernatant was collected in a fresh Eppendorf tube as soluble protein fraction. The remaining pellet was resuspended by sonication in 6 m urea buffer (6 m urea, 3% SDS, and RIPA), followed by centrifugation at 12,000 × *g* for 20 min at 4°C and stored at −80°C. For whole-cell lysate, tissue samples were homogenized in hot 1% SDS buffer and centrifuged at 12,000 × *g* for 20 min at room temperature, and the supernatant was collected in a fresh Eppendorf tube. Bradford (Sigma-Aldrich) or BCA (Bio-Rad) protein assay was performed for protein concentration estimation, and samples were prepared in 1× SDS loading buffer. Samples (20 µg) were loaded and separated by 8% SDS polyacrylamide gel electrophoresis followed by transfer on a methanol-charged PVDF membrane. Blocking was done with 4% BSA or 4% nonfat skimmed milk for 1 h at room temperature, followed by suitable primary antibody (Table 2) incubation. The next day PVDF membranes were washed with PBST (PBS, 0.1% Tween 20) 3× for 5 min followed by suitable secondary antibody (Table 2) incubation for 1 h at room temperature. After the secondary antibody incubation, the blots were washed in PBST 3× and developed in Bio-Rad chemiluminescent developer with ECL reagent (Bio-Rad). Immunoblots were acquired at appropriate exposure and further used for the quantitative analysis.

### Nerve root sectioning and axon caliber measurement

Dorsal and ventral root samples were collected as described above in 4% PFA and stored in 1% glutaraldehyde at 4°C. Toluidine blue staining was performed to visualize the axons. Briefly, tissue was washed 3× in NaHPO_4_, pH 7.4, for 5 min and incubated in 1% osmium tetroxide (Electron Microscopy Sciences) for 90 min at room temperature. After incubation, tissue was dehydrated in alcohol (50, 70, 90, and 100%), 50/50 ethanol/acetone, and acetone for 5 min each, and then 50/50 acetone/epoxy resin for 1 h at room temperature. Tissue was embedded in epoxy resin (TAAB 812, DDSA, NMA, and DMP30; Electron Microscopy Sciences) overnight at room temperature. The next day, the tissue was transferred into fresh epoxy and incubated at 60°C for 24–48 h; 1 µm semithin dorsal and ventral sections were obtained by ultramicrotome (Leica Ultra Cut E) and stained with toluidine blue for 1 min, then washed with Milli-Q. Slides were mounted with DPX paramount media and visualized under a light microscope (Leica DM5000 B) with 20× and 100× objectives.

### Electron microscopy

Ventral root samples were prepared in epoxy resin as explained above. Briefly, the tissue was postfixed in 1% osmium tetroxide for 90 min followed by alcohol dehydration (50, 70, 90, and 100%), propylene oxide, at room temperature, followed by embedding in epoxy resin. Ultrathin 90 nm sections were obtained, and images were captured in a JEOL 1230 Transmission Electron Microscope.

### Image analysis

Image analysis was performed using Fiji ImageJ software. NF immunofluorescence intensity in the brain was measured by adjusting adequate threshold, and total mean fluorescence intensity was taken for the entire field in the brain. A similar parameter was used for glial fibrillary acidic protein (Gfap)-Iba1 analysis in the brain and spinal cord. For the spinal cord lumbar region, image analysis was performed as described previously (Dutta et al., 2020). Neurons bigger than 250 µm^2^ were considered for quantification. Briefly, NeuN was marked with a hand tool in ImageJ to cover the entire cell body area. Integrated density of the protein of interest was taken by switching to the next channel. Nissl stain counting was done using particle analysis in ImageJ with a size-based filter above 50 and 150 µm^2^ for the brain and spinal cord, respectively. Axon caliber quantification was performed as explained above (Picher-Martel et al., 2019), and the total number of axons was quantified using the particle analysis plug-in. For immunoblot, band intensities were quantified by ImageJ. The value was normalized by a loading control (Actin or tubulin).

All statistical analyses were performed using GraphPad Prism 9.1.1.225 software. Comparison among multiple groups was done using one-way ANOVA with Bonferroni's correction *post hoc* test; a *p* value <0.05 was considered significant.

## Results

### Young gan^−/−^;TgPer mice exhibit symptom phenotypes and large accumulations of neurofilaments

**Figure 2.**
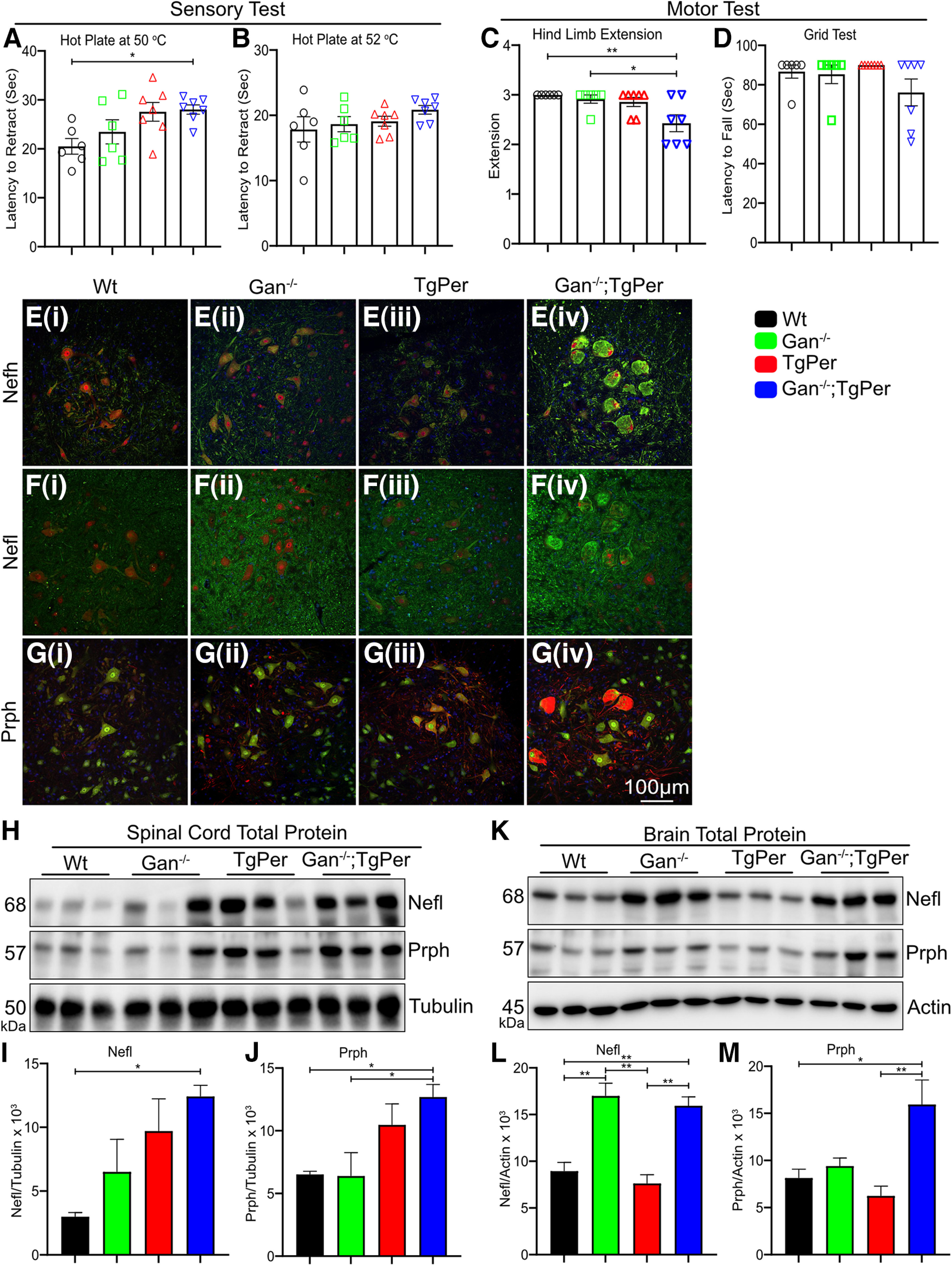
Symptom phenotypes and neurofilament accumulations exhibited by 3-month-old Gan^−/−^;TgPer mice. ***A***, ***B***, Graphs show the quantification for the hot plate sensory test at 50°C and 52°C, respectively. The Gan^−/−^;TgPer mice exhibit significant increase in latency to retract their paws from the hot plate at 50°C (***A***), in comparison with WT mice, *p = 0.0396 (WT vs Gan^−/−^;TgPer). ***C***, ***D***, Graphs show the quantification for the hindlimb extension and grid test, respectively. The Gan^−/−^;TgPer mice exhibited a significant reduction in hindlimb extension (***C***) **p = 0.0093 (WT vs Gan^−/−^;TgPer) in comparison with WT and Gan^−/−^ mice, *p = 0.0325 (Gan^−/−^ vs Gan^−/−^;TgPer). The grid test (***D***) did not show significant difference among the groups; *n* = 6–7 mice per group for sensory and motor tests. ***Ei***–***Giv***, Images represent immunofluorescence staining for Nefh, Nefl, and Prph, respectively. Colors green, red, and blue represent Nefh or Nefl, NeuN, and DAPI for images ***Ei–iv*** and ***Fi–iv***. Colors green, red, and blue represent NeuN, Prph, and DAPI for image ***G***. Original magnification 25×. Scale bar, 100 µm. Unlike WT mice (***Ei***, ***Fi***, ***Gi***), Gan^−/−^ mice (***Eii***, ***Fii***, ***Gii***), and TgPer mice (***Eiii***, ***Fiii***, ***Giii***), the Gan^−/−^;TgPer (***Eiv***, ***Fiv***, ***Giv***) exhibited large accumulations of neurofilament proteins Nefh (green), Nefl (green), and Prph (red) at 3 months of age. ***H***, Image represents total protein extract Western blot of Nefl, Prph, and Tubulin in the spinal cord. ***I***, ***J***, Graphs show quantification for the Nefl and Prph in the spinal cord. The Gan^−/−^;TgPer mice exhibit a significant increase in levels of Nefl and Prph proteins in comparison with WT and Gan^−/−^ mice. Nefl, *p = 0.0288 (WT mice vs Gan^-/^;TgPer); Prph, *p = 0.0486 (WT mice vs Gan^−/−^;TgPer) and *p = 0.0443 (Gan^−/−^ mice vs Gan^−/−^;TgPer). ***K***. Image represents Western blots for Nefl, Prph, and actin in total protein extracts from the brain. ***L***, ***M***, Graphs show quantification for Nefl and Prph in the brain. The Gan^−/−^;TgPer mice exhibited a significant increase in levels of Nefl in comparison with WT mice and TgPer mice in ***L***; **p = 0.0070 (WT mice vs Gan^−/−^;TgPer) and **p = 0.0024 (TgPer mice vs Gan^−/−^;TgPer). Prph protein levels of Gan^−/−^;TgPer mice in the brain were also significantly increased in comparison with WT mice (*p = 0.0295) and TgPer mice (*p = 0.0091); *n* = 3 mice in each group. Data represent ± SEM; one-way ANOVA with Bonferroni's multiple-comparisons *post hoc* test.

The double transgenic mice Gan^−/−^;TgPer were generated by crossing the Gan^−/−^ mice (Dequen et al., 2008) with transgenic mice overexpressing Prph (TgPer mice; Beaulieu et al., 1999) according to the scheme shown in Figure 1*A*. The mouse genotypes were determined by PCR analysis of tail DNA (Fig. 1*B*). After successfully generating the Gan^−/−^;TgPer mice along with WT, Gan^−/−^, and TgPer mice, we conducted a series of preliminary behavior, motor tests, and immunochemical assay to assess the early onset phenotypes and pathology of GAN disease at 3 months of age. To evaluate the sensory phenotypes, we performed the hot plate test at 50°C and 52°C (Fig. 2*A*,*B*). The Gan^−/−^;TgPer (28 s, *n* = 7 mice) mice exhibited a significant increase in the latency to retract the paws from the hot plate at 50°C in comparison with WT (20 s, *n* = 6); *p* value 0.0396 (Fig. 2*A*). In contrast, at 52°C the animals did not show any difference in retracting the paws from the plate (Fig. 2*B*). Furthermore, to assess the motor function, hindlimb extension and grid tests were performed (Fig. 2*C*,*D*). The Gan^−/−^;TgPer mice exhibited a significant reduction in hindlimb extension (2.4, *n* = 7 mice) in comparison with WT mice (3, *n* = 6) and Gan^−/−^ mice (2.9, *n* = 6); *p* values 0.0093 and 0.0325 (Fig. 2*C*). The Gan^−/−^;TgPer mice also exhibited frequent hindlimb clasping, a phenotype of stress/anxiety, that was not observed in Gan^−/−^, TgPer, and WT mice (data not shown). Nonetheless, the grid test did not yield a significant difference among different genotypes (Fig. 2*D*).

Further, immunostaining in the lumbar region of the spinal cord revealed massive IF accumulations with prominent immunofluorescence staining for Nefh, Nefl, and Prph in the Gan^−/−^;TgPer mice (Fig. 2*E–G*). Such IF accumulations were not seen in WT, Gan^−/−^, and TgPer mice. The spinal neurons of Gan^−/−^;TgPer were large and swollen in shape (Fig. 2*Eiv*,*Fiv*,*Giv*), whereas the morphology of the spinal neurons of WT, Gan^−/−^, and TgPer remained intact. Furthermore, Western blotting with total protein extract from the spinal cord and the brain was performed to assess the levels of Prph species along with Nefl in 3-month-old Gan^−/−^;TgPer mice when compared with age-matched WT, Gan^−/−^, and TgPer mice (Fig. 2*H*,*K*). Immunoblot quantification of spinal cord revealed a significant increase in levels of Nefl (about six folds) protein in the Gan^−/−^;TgPer mice in comparison with WT mice (Fig. 2*I*). As expected, the Gan^−/−^;TgPer mice also exhibited a significant increase in levels of Prph in the spinal cord (∼2.5 folds) in comparison with WT and Gan^−/−^ mice (Fig. 2*H*,*J*). Similarly, Western blotting with brain extracts also revealed significant increases in levels of Nefl (about twofold) and of Prph (about twofold) proteins in Gan^−/−^;TgPer mice in comparison with brain extracts from WT and TgPer mice (Fig. 2*K*,*L*). These results indicated that the formation of abnormal IF accumulations starts at an early age in Gan^−/−^;TgPer mice (Fig. 2*E–M*). The results also suggested that a threshold level of Prph is required to trigger the disorganization of the NF network. This NF pathology in the Gan^−/−^;TgPer mice was associated with the development of sensory and motor deficits (Fig. 2*A–D*) at a young age.

### Cognitive and motor defects exhibited by gan^−/−^;TgPer mice during aging

**Figure 3.**
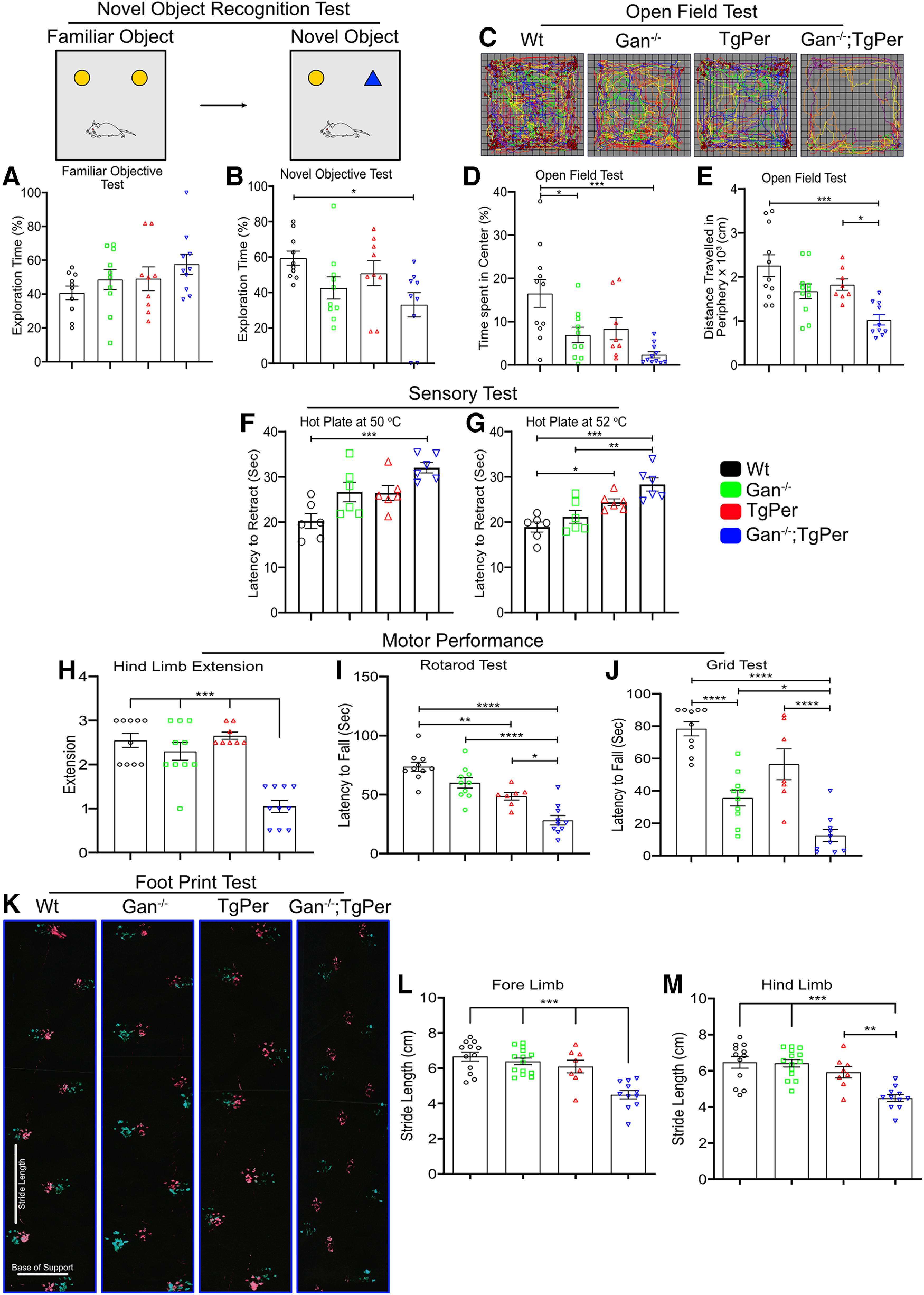
Gan^−/−^;TgPer mice exhibit cognitive and motor decline at the age of 12 months. ***A***, ***B***, The quantification for novel object recognition test. The Gan^−/−^;TgPer mice exhibited a significant decrease in total percentage of exploration time on novel object in comparison with WT mice, *p = 0.021. ***C–E***, The heat map of the open field test of WT, Gan^−/−^, TgPer, and Gan^−/−^;TgPer mice (***C***), whereas ***D*** and ***E*** show the open field quantification for percentage of time spent in the center and total distance traveled in the periphery (cm), respectively. The Gan^−/−^;TgPer mice exhibited a significant reduction in the percentage of time spent in the center in comparison with WT mice (***D***; ***p = 0.0003). The total distance traveled in the periphery by Gan^−/−^;TgPer mice was also significantly reduced in comparison with WT mice (***p = 0.0001) and TgPer mice (*p = 0.0362), as shown in ***E***. ***F***, ***G***, Graphs show the quantification for the hot plate sensory test at 50°C and 52°C, respectively. The Gan^−/−^;TgPer mice exhibited a significant increase in the latency to retract their paws at 50°C and 52°C; hot plate test, ***p = 0.0005 (WT vs Gan^−/−^;TgPer) at 50°C and ***p = 0.0001 (WT vs Gan^−/−^;TgPer); **p = 0.0028 (Gan^−/−^ vs Gan^−/−^;TgPer) at 52°C. TgPer mice also showed a significant increase in the latency to retract the paw at 52°C in comparison with WT mice, *p = 0.0258 (***G***). ***H***–***J***, Graphs show the quantitative analysis of hindlimb extension, rotarod, and grid tests, respectively. Hindlimb extension was significantly reduced for the Gan^−/−^;TgPer mice in comparison with WT, Gan^−/−^ and TgPer mice ***p = 0.0001; ***H***). The latency to fall from rotarod was significantly reduced for the Gan^−/−^;TgPer mice in comparison with WT (****p = 0.0001), Gan^−/−^, and TgPer mice (*p = 0.0117; ***I***). Similarly, the grid test revealed significant reduction in latency to fall for Gan^−/−^;TgPer mice in comparison with WT, Gan^−/−^, and TgPer mice (***J***); ****p = 0.0001, *p = 0.0213 (WT vs Gan^−/−^;TgPer; TgPer vs Gan^−/−^;TgPer, Gan^−/−^ vs Gan^−/−^;TgPer). ***K***, Image represents the results of the footprint test. Colors green and red indicate the forelimbs and hindlimbs of mice, respectively. Graphs (***L***, ***M***) show quantification for the stride length of forelimbs and hindlimbs. The Gan^−/−^;TgPer mice exhibit a significant reduction in the stride length of the forelimbs and hindlimbs in comparison with WT mice (***p = 0.0001), Gan^−/−^ mice, and TgPer mice (***p = 0.0009); *n* = 6–14 mice per group. Data indicate ± SEM; one-way ANOVA with Bonferroni's multiple-comparisons *post hoc* test.

We generated cohorts of mice of different genotypes (WT, Gan^−/−^, TgPer, and Gan^−/−^;TgPer) and performed a series of behavioral analyses at 12 months of age. The Gan^−/−^;TgPer mice exhibited cognitive impairment based on the novel object recognition test (Fig. 3*A*,*B*). The Gan^−/−^;TgPer mice spent only 33% of time (*n* = 10) with the novel object in comparison with WT mice (*n* = 10) that spent 59% of time with the new object. The difference of 26% is significant (*p* = 0.021). The results of the novel object test suggest that Gan^−/−^;TgPer mice have a reduction in temporal-lobe-based memory function at the age of 12 months.

The mice were also subjected to the open-field test, which consisted of allowing mice to move freely in an open chamber for 30 min under the dark condition, and their movements were recorded in six different cycles of 5 min each to calculate the percentage of time spent in the center of the chamber. The heat map generation of Gan^−/−^;TgPer indicated severe anxiety stress (Fig. 3*C*). The Gan^−/−^;TgPer mice exhibited a significant reduction in the percentage of time spent in the center (2.3%, *n* = 11) in comparison with WT (16.5%, *n* = 11) mice; *p* = 0.0003 (Fig. 3*D*). The Gan^−/−^ mice also showed a significant change (6.9%, *n* = 10) in comparison with WT mice; *p* = 0.0247. Further, we analyzed the average traveled distance in the periphery of the chamber. The Gan^−/−^;TgPer mice showed a significant reduction of total traveled distance in the periphery (1027 cm, *n* = 10) in comparison with WT mice (2258 cm, *n* = 11) and TgPer mice (1826 cm, *n* = 8); *p* values 0.0001 and 0.0362, respectively (Fig. 3*E*).

A hot plate test at 50°C and 52°C was also conducted to assess sensory deficits at 12 months of age (Fig. 3*F*,*G*). The Gan^−/−^;TgPer mice exhibited a significant increase in the latency to retract their paws at 50°C (32 s, *n* = 6) in comparison with WT mice (20 s, *n* = 6); *p* = 0.0005 (Fig. 3*F*). Moreover, at 52°C the Gan^−/−^;TgPer mice exhibited a significant increase in latency to retract their paws from the plate (28 s, *n* = 6) in comparison with WT mice (18 s, *n* = 6) and Gan^−/−^ mice (21 s, *n* = 6); *p* values 0.0001 and 0.0028, respectively (Fig. 3*G*). These results suggest that Gan^−/−^;TgPer mice exhibited sensory deficits at 12 months of age.

The motor performance of Gan^−/−^;TgPer mice at 12 months of age was assessed by performing hindlimb extension, rotarod, grid strength, and footprint tests (Fig. 3*H–J*). The Gan^−/−^;TgPer mice exhibited a significant reduction of hindlimb extension (score 1.05, *N* = 10) in comparison with WT mice (score 2.55, *N* = 10), Gan^−/−^ mice (score 2.30, *N* = 10), and TgPer mice (score 2.65, *N* = 8). Thus, the reduction in hindlimb extension score for the Gan^−/−^;TgPer mice was ∼2.5-fold; *p* = 0.0001 (Fig. 3*H*). With the rotarod test, the Gan^−/−^;TgPer mice exhibited a sharp decline in latency to fall (28 s, *N* = 10) in comparison with WT mice (74 s, *N* = 10), Gan^−/−^ mice (60 s, *N* = 10) and TgPer mice (49 s, *N* = 7) mice. So the Gan^−/−^;TgPer mice performed the rotarod test with a threefold decrease in latency to fall in comparison with WT mice; *p* = 0.0013 (Fig. 3*I*). The grid hang test was also conducted to assess muscle strength. The Gan^−/−^;TgPer mice fell down after 13 s (*n* = 10) in comparison with 78 s for WT mice (*n* = 10), 36 s for Gan^−/−^ mice (*n* = 10), and 56 s for TgPer mice (*n* = 7; Fig. 3*J*). These combined results indicate significant strength loss for the Gan^−/−^;TgPer mice at 12 months of age.

Further, a footprint test was also performed to assess gait impairment. The stride lengths of the forelimbs and hindlimbs were measured (Fig. 3*K*). The forelimb stride length of Gan^−/−^;TgPer mice was significantly reduced (4.5 cm, *n* = 11) in comparison with the stride length of WT mice (6.7 cm, *n* = 12), Gan^−/−^ mice (6.9 cm, *n* = 14), and TgPer mice (6.1 cm, *N* = 8; Fig. 3*L*). Similarly, the hindlimb stride length of Gan^−/−^;TgPer mice (4.5 cm, *n* = 11) was significantly reduced in comparison with WT mice (6.5 cm, *n* = 12), Gan^−/−^ mice (6.4 cm, *n* = 14), and TgPer mice (5.9 cm, *n* = 8; Fig. 3*M*). The combined results suggest that the Gan^−/−^;TgPer mice, during aging, exhibit overt cognitive and sensory motor dysfunction phenotypes, which are reminiscent of human GAN disease.

### NF disorganization in the spinal cord and brain

**Figure 4.**
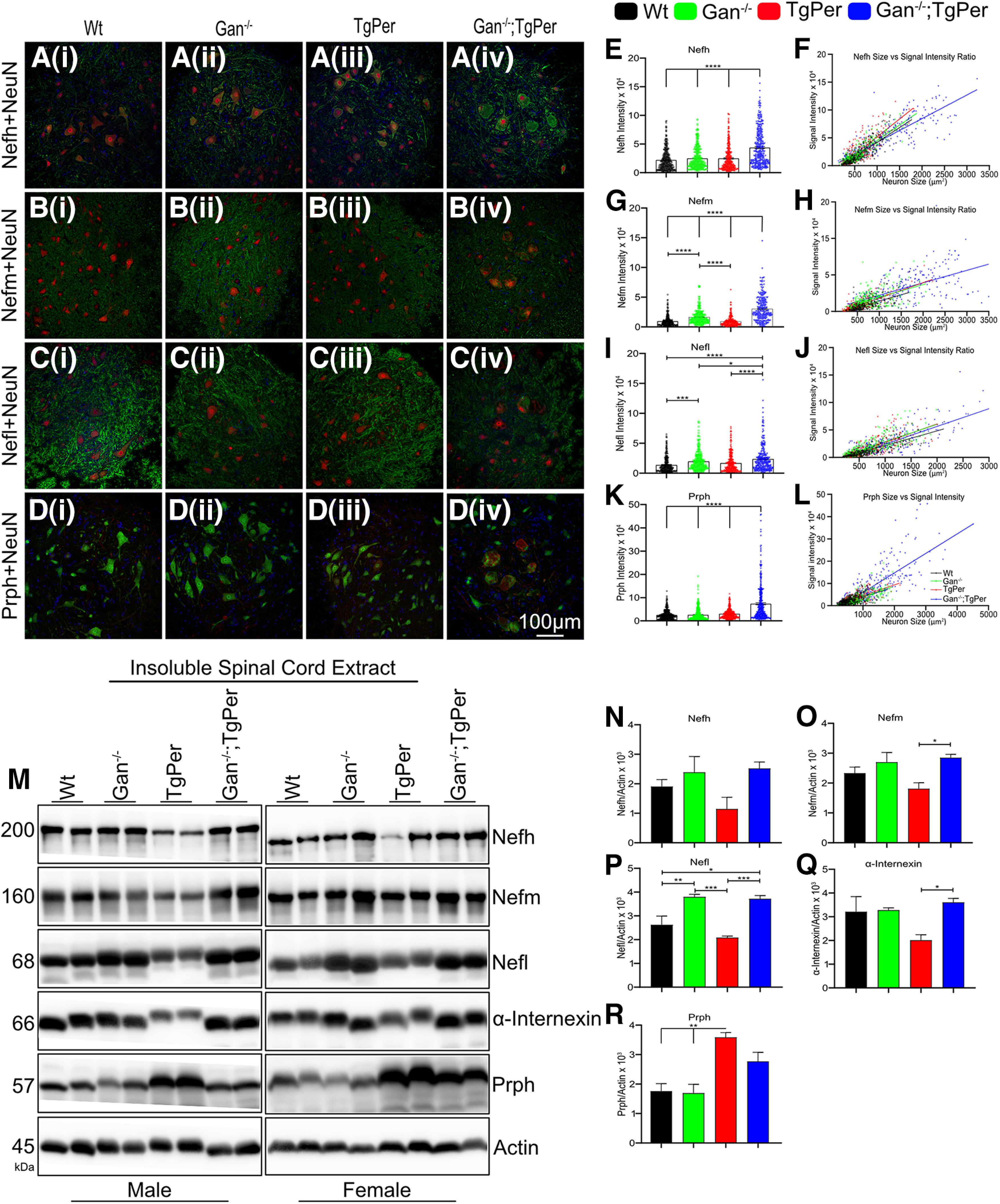
NF accumulations in spinal neurons of 12-month-old Gan^−/−^;TgPer mice. ***Aiv–Div***, Images show immunofluorescence staining of Nefh, Nefm, Nefl, and Prph, respectively, in the spinal cord of WT, Gan^−/−^, TgPer, and Gan^−/−^;TgPer mice. Large accumulations of NFs caused swelling of spinal neurons in Gan^−/−^;TgPer mice (***Aiv***, ***Biv***, ***Civ***, ***Div***). Original magnification 20×. Scale bar, 100 µm. Green represents Nefh, Nefm, and Nefl; red represents NeuN; and blue represents DAPI (***Ai–iv to Ci–iv***). Colors green, red, and blue represent NeuN, Prph, and DAPI for ***Di–iv***. ***E***, ***G***, ***I***, ***K***, Graphs represent quantitative analysis of signal intensity for Nefh, Nefm, Nefl, and Prph. The Gan^−/−^;TgPer mice exhibited a significant increase in levels of NF proteins and Prph signals in comparison with WT, Gan^−/−^, and TgPer mice. The signal intensities of spinal neurons larger than 250 µm^2^ were taken from at least six spinal cord sections; *n* = 3 mice per group. Nefh, ****p = 0.0001 (WT vs Gan^−/−^;TgPer mice, Gan^−/−^ vs Gan^−/−^;TgPer mice, TgPer vs Gan^−/−^;TgPer mice); Nefm, ****p = 0.0001 (WT vs Gan^−/−^;TgPer mice, Gan^−/−^ vs Gan^−/−^;TgPer mice, TgPer vs Gan^−/−^;TgPer mice); Nefl, ****p = 0.0001 (WT vs Gan^−/−^;TgPer mice, TgPer vs Gan^−/−^;TgPer mice), *p = 0.0331 (Gan^−/−^ vs Gan^−/−^TgPer mice); Prph, ****p = 0.0001 (WT vs Gan^−/−^;TgPer mice, Gan^−/−^ vs Gan^−/−^;TgPer mice, TgPer vs Gan^−/−^;TgPer mice). The Gan^−/−^ mice also showed a significant increase in levels of Nefm and Nefl against WT and TgPer mice (***G***, ***I***); Nefm, ****p = 0.0001 (WT vs Gan^−/−^ mice, TgPer vs Gan^−/−^mice); Nefl, ***p = 0.0002 (WT vs Gan^−/−^ mice). ***F***, ***H***, ***J***, ***L***, Graphs indicate a direct correlation between the size of spinal neurons and the signal intensity for immunostaining of Nefh, Nefm, Nefl, and Prph, respectively. The Gan^−/−^;TgPer mice exhibited spinal neurons of larger size and with higher levels of NF protein signals. ***M***, Image represents the Western blots of insoluble spinal cord extract from WT, Gan^−/−^, TgPer, and Gan^−/−^;TgPer mice. ***N***–***Q***, The samples from Gan^−/−^;TgPer mice had a significant increase in levels of Nefm, Nefl, and α-internexin, respectively; Nefm, *p = 0.0392 (TgPer vs Gan^−/−^;TgPer mice); Nefl, *p = 0.0158, ***p = 0.0007 (WT vs Gan^−/−^;TgPer mice, TgPer vs Gan^−/−^;TgPer mice); α-internexin, *p = 0.0461 (TgPer vs Gan^−/−^;TgPer mice). The Gan^−/−^ mice also showed a significant increase in the levels of insoluble Nefl in comparison with WT and TgPer mice (***P***); Nefl, **p = 0.0099, ***p = 0.0004 (WT vs Gan^−/−^ mice, Gan^−/−^ vs TgPer mice). ***R***, A significant increase in the levels of insoluble Prph was also found in TgPer mice vs WT and Gan^−/−^ mice. Prph, **p = 0.0022, **p = 0.0016 (WT vs TgPer mice, Gan^−/−^ vs TgPer mice). It is noteworthy that the levels of Prph in samples from Gan^−/−^;TgPer mice were slightly lower than those from TgPer mice (***R***). The reduced Prph levels may reflect in part the loss of Prph-expressing spinal neurons at 12 months of age. For each group, two males and two females were analyzed; *n* = 4 mice in each group. Data indicate ± SEM; one-way ANOVA with Bonferroni's multiple-comparisons *post hoc* test.

The disorganization of NF network was sustained during aging in Gan^−/−^;TgPer mice. Spinal cord lumbar region (L4 and L5) samples were examined at microscopy from mice at 12 months of age (Fig. 4). Immunofluorescence microscopy revealed increased levels of Nefh, Nefm, Nefl, and Prph in Gan^−/−^;TgPer when compared with WT, Gan^−/−^, and TgPer mice. The integrated density of spinal neurons larger than 250 µm^2^ was analyzed by ImageJ software. The signal intensity of 300–350 neurons from each group was measured. The signal density of Nefh, Nefm, Nefl, and Prph was significantly increased by 2-, 3-, 2.5-, and 5-fold, respectively (Fig. 4*E*,*G*,*I*,*K*). Whereas spinal neurons in WT, Gan^−/−^, and TgPer mice were normal in morphology, the Prph overexpression in Gan^−/−^;TgPer mice led to neurons with a giant balloon shape. We found that the larger size of spinal neurons correlated with the higher signal intensity of the respective NF protein (Fig. 4*F*,*H*,*J*,*L*). The data suggest that a buildup of Prph and NF proteins led to large swellings of spinal neurons in Gan^−/−^;TgPer mice.

**Figure 5.**
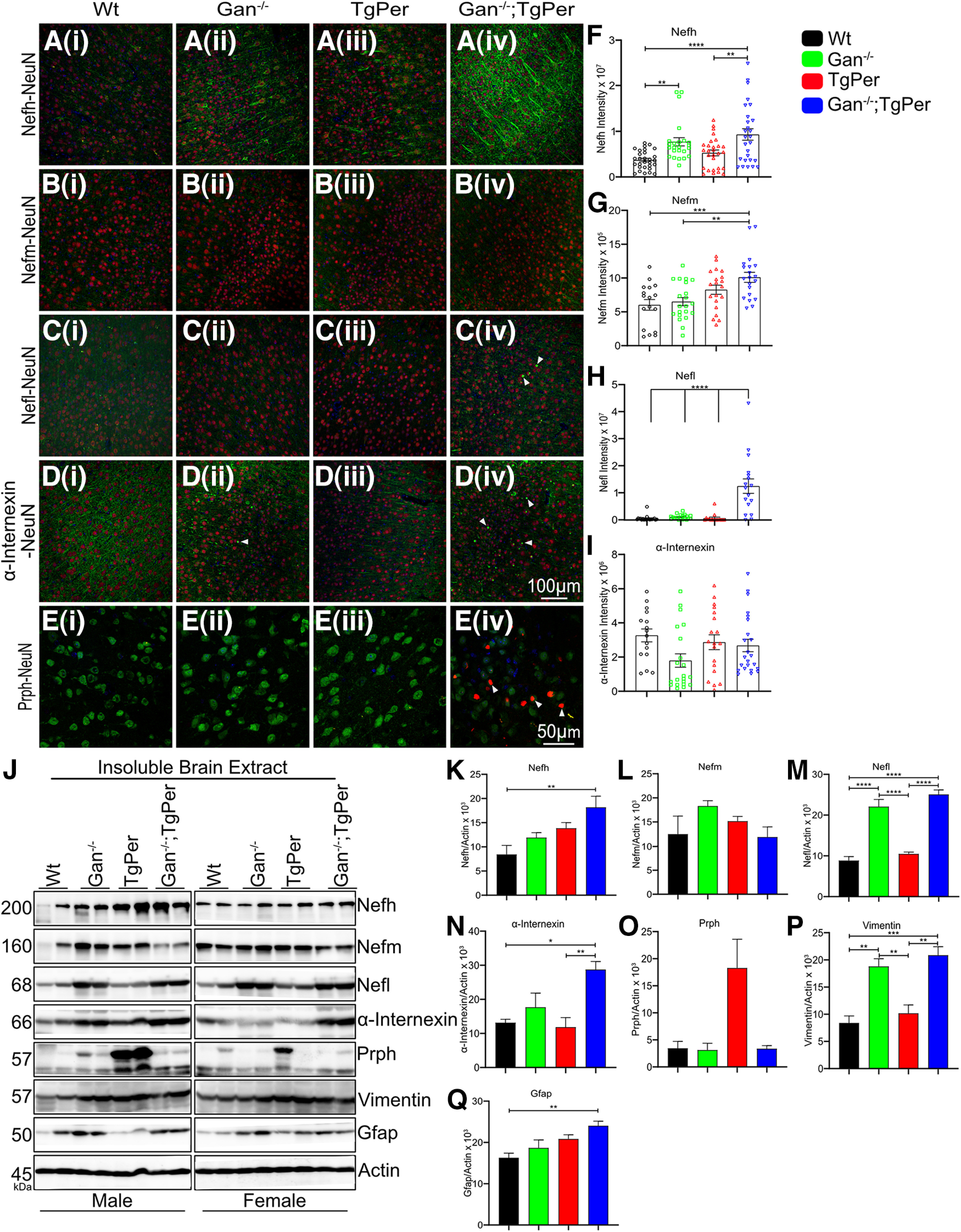
Abnormal NF accumulations in the brain cortex of Gan^−/−^;TgPer mice at 12 months of age. ***Ai–Eiv***, Images represent the immunostaining for Nefh, Nefm, Nefl, α-internexin, and Prph, respectively, in the cortex of WT, Gan^−/−^, TgPer, and Gan^−/−^;TgPer mice. Original magnification of 25× for NF proteins and 63× for Prph. Scale bars: 100 µm for Ai-Div images, 50 µm for Ei-iv images. Green represents Nefh, Nefm, Nefl, and α-internexin, whereas red represents NeuN and blue represents DAPI (***Ai–Div***). ***Ei–iv***, Colors green, red, and blue represent NeuN, Prph, and DAPI, respectively. The Gan^−/−^;TgPer mice exhibit massive accumulations of Nefh in neuronal perikarya and neurites (***Aiv***) as well as abundant inclusion bodies composed of Nefl, α-internexin, and Prph proteins. Arrowheads in ***Civ***, ***Div***, ***Eiv*** indicate NF inclusion bodies in Gan^−/−^;TgPer mice. ***F–I***, Graphs represent the quantitative analysis of signal intensity for Nefh, Nefm, Nefl, and α-internexin. There are significant increases in the signal intensity for Nefh, Nefm, and Nefl in the brain of Gan^−/−^;TgPer mice (***F***, ***G***, ***H***). At least six brain sections per mice with three mice per group were used for quantification; *n* = 3 mice in each group. Nefh, **p = 0.0053; ****p = 0.0001; **p = 0.0078 (WT vs Gan^−/−^;TgPer mice, TgPer vs Gan^−/−^;TgPer mice, WT vs Gan^−/−^ mice); Nefm, **p = 0.0023; ***p = 0.0009 (Gan^−/−^ vs Gan^−/−^;TgPer mice, WT vs Gan^−/−^;TgPer mice); Nefl, ****p = 0.0001 (WT vs Gan^−/−^;TgPer mice, Gan^−/−^ vs Gan^−/−^;TgPer mice, TgPer vs Gan^−/−^;TgPer mice). ***J***, Image represents Western blots for the insoluble brain extracts from WT, Gan^−/−^, TgPer, and Gan^−/−^;TgPer mice. ***K***, ***L***, ***M***, ***N***, ***O***, ***P***, ***Q***, Graphs reveal significant increase in levels of insoluble Nefh, Nefl, α-internexin, vimentin, and Gfap in Gan^−/−^;TgPer mice. Nefh, **p = 0.0088 (WT vs Gan^−/−^;TgPer mice); Nefl, ****p = 0.0001 (WT vs Gan^−/−^;TgPer mice, TgPer vs Gan^−/−^;TgPer mice); α-internexin *p = 0.0123, **p = 0.0068 (WT vs Gan^−/−^;TgPer mice, TgPer vs Gan^−/−^;TgPer mice); vimentin ***p = 0.0003, **p = 0.0014 (WT vs Gan^−/−^;TgPer mice, TgPer vs Gan^−/−^;TgPer mice); Gfap, **p = 0.0093 (WT vs Gan^−/−^;TgPer mice). The Gan^−/−^ mice also showed significant increases in levels of Nefl and vimentin compared with WT and TgPer mice (***D***, ***G***). Nefl, ****p = 0.0001 (WT vs Gan^−/−^ mice, TgPer vs Gan^−/−^ mice); vimentin **p = 0.0017, **p = 0.0076 (WT vs Gan^−/−^ mice, TgPer vs Gan^−/−^ mice). The levels of Nefm and Prph proteins did not change significantly among the groups (***C***, ***F***). Two male and two female mice were considered for each group; *n* = 4 mice in each group. Data indicate ± SEM, one-way ANOVA with Bonferroni's multiple-comparison *post hoc* test.

Neurofilament disorganization was also detected in the brain of Gan^−/−^;TgPer mice (Fig. 5). We have conducted immunofluorescence microscopy of the cerebral cortex from 12 month-old mice to monitor the changes in levels and distribution of NF proteins (Nefh, Nefm, Nefl, α-internexin, and Prph). The mean fluorescence intensity was acquired by ImageJ software to measure the increased levels of immunostaining in the brain. In the brain cortex of Gan^−/−^;TgPer mice, there was a significant increase in levels of NF proteins (Nefh, Nefm, Nefl, and α-internexin) in comparison with WT, Gan^−/−^, and TgPer mice (Fig. 5*F–I*). The immunofluorescence staining for Nefh was increased by about twofold in the cortex of Gan^−/−^;TgPer mice (Fig. 5*F*) in comparison with WT and TgPer mice. The Nefh immunostaining protein was very dense in the neuronal cell bodies and axons in the cortex (Fig. 5*Aiv*). It is noteworthy that the signal intensity for Nefl in the cortex of Gan^−/−^;TgPer mice was significantly increased by about sixfold in comparison with WT, Gan^−/−^, and TgPer mice (Fig. 5*H*). Unlike Nefh, the Nefl protein accumulated mainly in neuronal IF inclusion bodies in the cortex (Fig. 5*Civ*, arrowheads). Moreover, immunofluorescence microscopy revealed an abundance of α-internexin accumulations (Fig. 5*Div*, arrowheads) as well as Prph accumulations (Fig. 5*Eiv*, arrowheads) in the cortex of Gan^−/−^;TgPer mice. It is noteworthy that large inclusion bodies containing Prph were absent in the brain of WT, Gan^−/−^, and TgPer mice (Fig. 5*Ei*,*ii*,*iii*).

**Figure 6.**
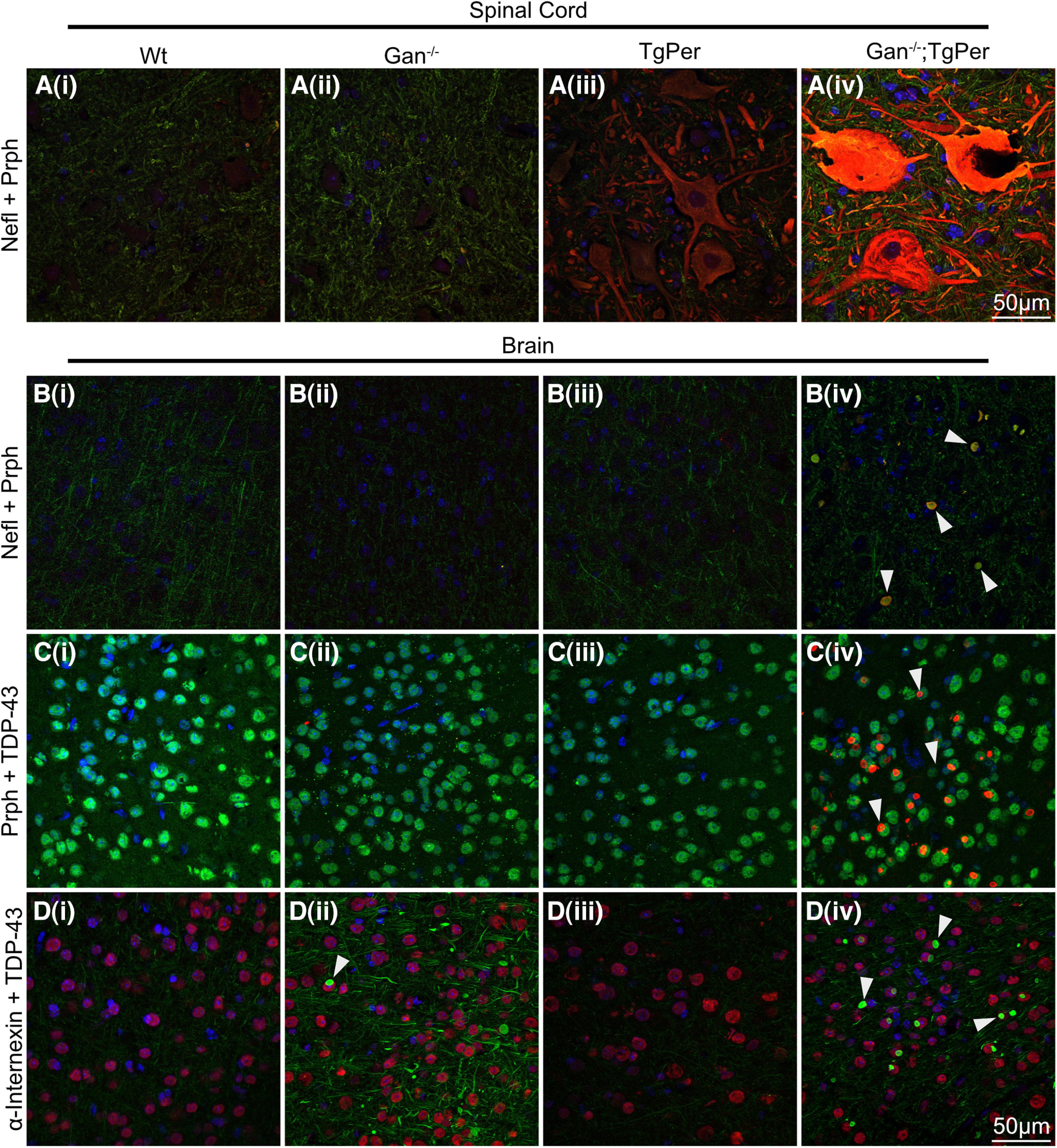
Colocalization of NF proteins, but not TDP-43, with Prph inclusions in the spinal cord and the cortex of Gan^−/−^;TgPer mice. ***Ai–iv to Bi–iv***, Images show immunofluorescence staining for Nefl and Prph in the spinal cord and brain, respectively. Colors green, red, and blue represent Nefl, Prph, and DAPI (***Ai–iv to Bi–iv***). White arrowheads point to the inclusion bodies made of Prph with NF proteins in ***Biv***, which shows the colocalization of Nefl with Prph inclusion bodies. ***Ci–iv to Di–iv***, Images show immunofluorescence staining in cortical neurons for Prph and TDP-43, and α-internexin + TDP-43, respectively. Colors green, red, and blue represent TDP-43, Prph, and DAPI for ***Ci–iv***. Colors green, red, and blue represent α-internexin, TDP-43, and DAPI, respectively, for ***Di–iv***. ***Civ*** and ***Div*** reveal that TDP-43 did not colocalize with inclusion bodies (arrowheads) composed of Prph and α-internexin in the brain of Gan^−/−^;TgPer mice. Original magnification 63×. Scale bar, 50 µm.

Coimmunostaining of Nefl and Prph was performed in the spinal cord and brain of mice at the age of 12 months (Fig. 6*Ai–iv*, *Bi–iv*). In the spinal cord and brain of Gan^−/−^;TgPer mice, there was colocalization of Nefl and Prph proteins in subsets of neurons overexpressing Prph. Nefl was coimmunostained with large Prph accumulations in spinal neurons of Gan^−/−^;TgPer mice (Fig. 6*Aiv*). Moreover, Gan^−/−^;TgPer mice exhibited in subset of cortical neurons inclusion bodies coimmunostained for Nefl and Prph (Fig. 6*Biv*, white arrowheads). In contrast, neither Gan^−/−^ mice nor TgPer mice developed significant IF inclusion bodies in the brain or spinal cord at 12 months of age. This suggests that a threshold level of Prph protein may be required to trigger disorganization of neuronal IFs.

The insoluble protein fractions from the spinal cord and brain cortex of mice at 12 months of age were subjected to SDS-PAGE and immunoblotting with antibodies against cytoskeletal proteins. As expected, the levels of NF proteins were increased in the spinal cord (Fig. 4) and cortex (Fig. 5) of Gan^−/−^;TgPer mice when compared with corresponding samples from WT and TgPer mice. It is noteworthy that at 12 months of age, the levels of Prph in the spinal cord (Fig. 4*R*) and brain (Fig. 5*O*) of Gan^−/−^;TgPer mice were lower than in corresponding samples from TgPer mice. This is in contrast with immunoblot results of spinal cord samples from mice at 3 months of age showing higher levels of Prph in the Gan^−/−^;TgPer mice when compared with TgPer mice (Fig. 2*H*,*J*). This apparent discrepancy in Prph levels is likely because of the loss of Prph-expressing neurons that occurred during aging. As described below (see Fig. 8), the Gan^−/−^;TgPer mice exhibited a significant loss (∼30%) of spinal motor neurons at 12 months of age.

The abnormal cytoplasmic accumulations of TDP-43 species in neurons constitute a hallmark of several neurodegenerative disorders including ALS and dementia (Neumann et al., 2006; Kumar et al., 2021). To examine the possibility that TDP-43 was a component of neuronal inclusion bodies in the Gan^−/−^;TgPer transgenic mice, we have also conducted coimmunostaining of TDP-43 with Prph or α-internexin (Fig. 6*C*,*D*). As shown in Figure 6, there was no colocalization of TDP-43 immunostaining with Prph (Fig. 6*Civ*) and α-internexin (Fig. 6*Div*) filamentous inclusions.

#### Neuroinflammation

**Figure 7.**
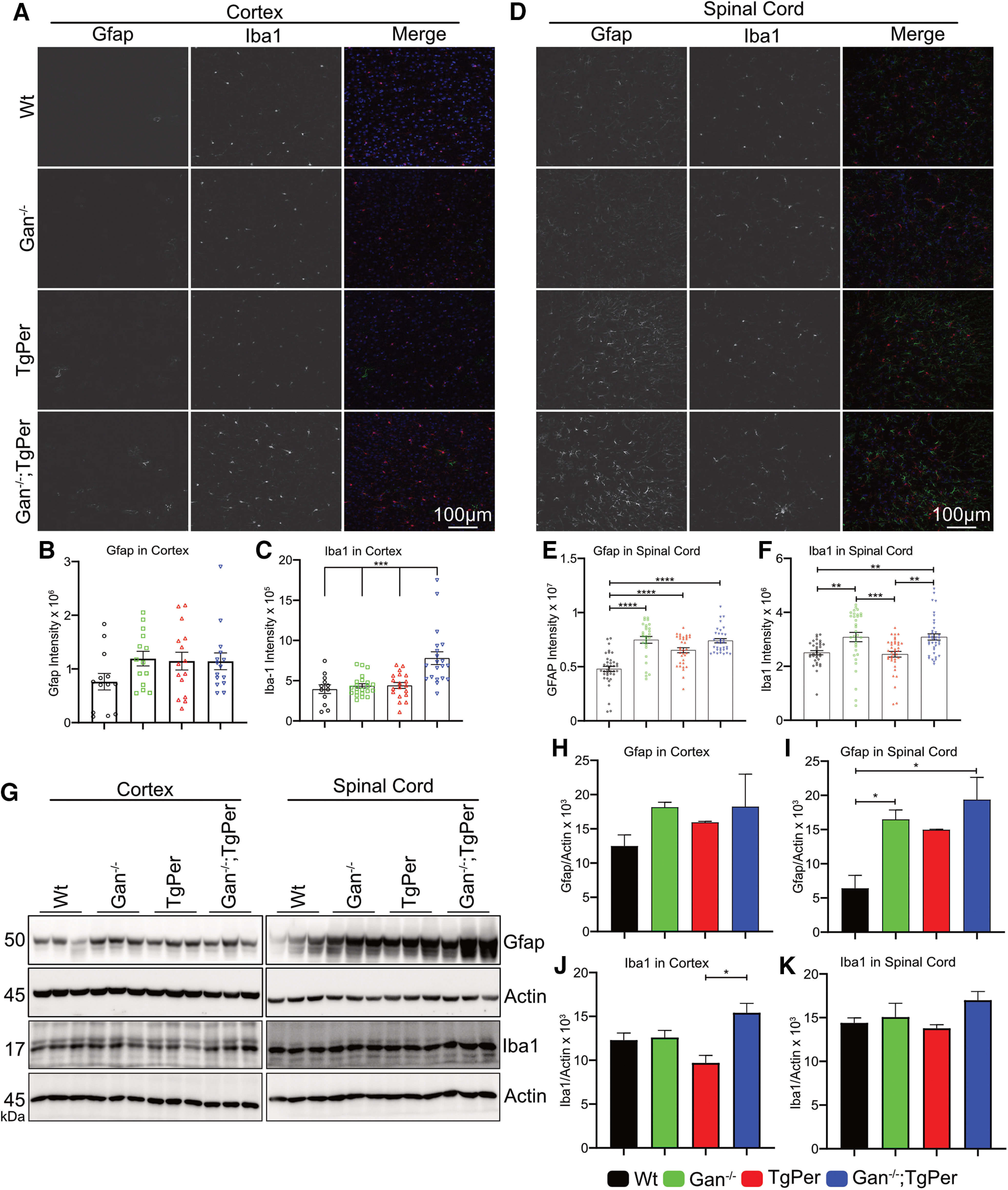
Neuroinflammation in the brain and spinal cord of Gan^−/−^;TgPer mice at the age of 12 months. ***A***, ***D***, Images represent the immunostaining for Gfap and Iba1 proteins in the brain and spinal cord. Original magnification 20×. Scale bar, 100 µm. Colors green, red, and blue represent Gfap, Iba1, and DAPI, respectively. At least six sections per mouse with three mice per group were considered for quantification (*n* = 3 mice per group). ***B***, Quantitative analysis of Gfap signal intensity in the cortex did not show a significant difference among different genotypes. ***E***, Gfap levels in the spinal cord were significantly increased in Gan^-/^mice, TgPer mice, and Gan^−/−^;TgPer mice in comparison with WT mice; ****p = 0.0001 (WT vs Gan^−/−^ mice, WT vs TgPer mice, WT vs Gan^−/−^;TgPer mice). ***C***, ***F***, The signal intensity of Iba1 in Gan^−/−^;TgPer mice was significantly higher in the brain and spinal cord when compared with the mouse groups. Iba1, ***p = 0.0001 (WT vs Gan^−/−^;TgPer mice, Gan^−/−^ vs Gan^−/−^;TgPer mice, TgPer vs Gan^−/−^TgPer mice) in the brain; **p = 0.0070 (WT vs Gan^−/−^;TgPer mice), **p = 0.0015 (TgPer vs Gan^−/−^;TgPer mice) in the spinal cord. The signal intensity of Iba1 from Gan^−/−^ mice was significantly higher than in WT and TgPer mice; **p = 0.0012 (WT vs Gan^−/−^ mice; ***p = 0.0002 (Gan^−/−^ vs TgPer mice). ***G***, Image shows Western blot for Gfap, Iba1, and actin in total extracts from the brain and spinal cord. ***I***, Quantitative analysis revealed significant increase in Gfap levels in the spinal cord of Gan^−/−^;TgPer and Gan^−/−^ mice in comparison with WT mice, graph; *p = 0.0113 (WT vs Gan^−/−^;TgPer mice); *p = 0.0457 (WT vs Gan^−/−^ mice). ***H***, Gfap levels in the brain did not change significantly in mice with different genotypes. ***J***, ***K***, Graphs show quantitative analysis of Iba1 in the brain and spinal cord, respectively. The Gan^−/−^;TgPer mice exhibited significant increase level of Iba1 in comparison with TgPer mice; *p = 0.0122. Iba1 levels in the spinal cord did not change significantly among the groups (***K***); *n* = 3 mice in each group. Data represent ± SEM; one-way ANOVA with Bonferroni's multiple-comparisons *post hoc* test.

We investigated whether the GAN-like pathology in Gan^−/−^;TgPer mice was associated with neuroinflammation. Immunofluorescence microscopy and Western blots for Iba1 and Gfap were conducted to assess microgliosis and astrogliosis, respectively. As shown in Figure 7, *A* and *B*, the Gfap signal intensity in the brain was similar among the different mouse genotypes (Fig. 7*A*,*B*). However, the Gfap signal in the spinal cord of Gan^−/−^;TgPer mice was significantly higher than in WT and TgPer mice (Fig. 7*D*,*E*). The signal intensity for Iba1 was increased in the brain by 1.5-fold in Gan^−/−^;TgPer mice in comparison with WT, Gan^−/−^, and TgPer mice (Fig. 7*A*,*C*). In the spinal cord, the Iba1 signal intensity in Gan^−/−^;TgPer mice was also significantly higher (1.5-fold) than in WT and TgPer mice (Fig. 7*D*,*F*). Accordingly, Western blots revealed higher levels of Gfap in the spinal cord of Gan^−/−^;TgPer mice when compared with WT, Gan^−/−^, and TgPer mice (Fig. 7*G*,*H*). Further, the Western blotting of Iba1 in the brain showed a significantly increased level in the Gan^−/−^;TgPer when compared with TgPer mice (Fig. 7*G*,*J*). The combined results suggest that the disease in this mouse model of GAN is associated with microgliosis and astrogliosis.

### Neurodegeneration in the brain and spinal cord

**Figure 8.**
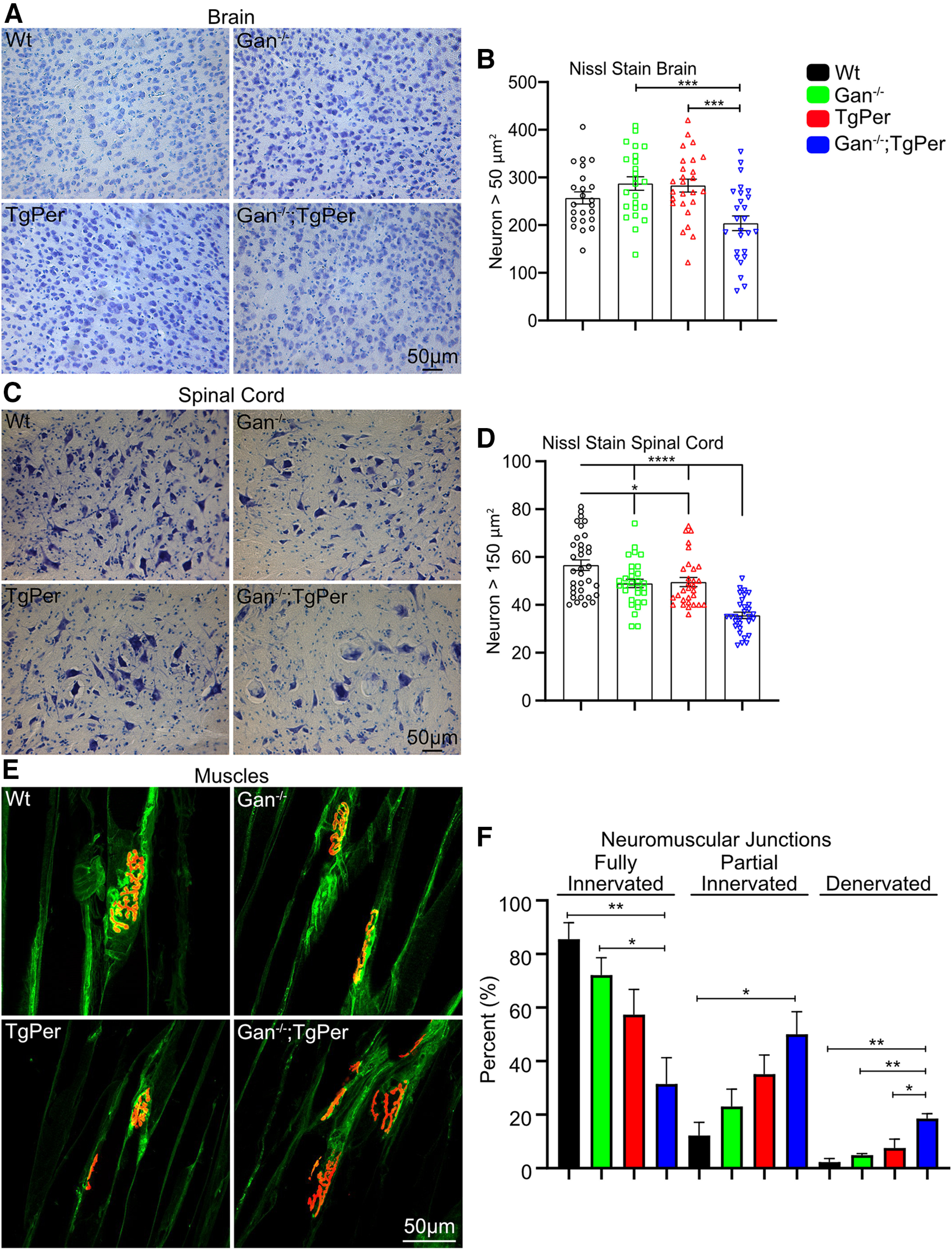
Neuronal loss and muscle denervation in 12-month-old Gan^−/−^;TgPer mice. ***A***, ***C***, Images show Nissl stain of the brain and spinal cord, respectively. Original magnification at 20×. Scale bar, 50 µm. Neurons larger than 50 and 150 µm^2^ were counted in the brain and spinal cord, respectively. ***B***, ***D***, Quantitative analysis revealed that the Gan^−/−^;TgPer mice exhibit significant loss of neurons in the brain and spinal cord at 12 month of age; *p* = *** 0.0004 (Gan^−/−^ vs Gan^−/−^;TgPer mice); ***p = 0.0007 (TgPer vs Gan^−/−^;TgPer mice) in the brain; ****p = 0.0001 (WT vs Gan^−/−^;TgPer mice, Gan^−/−^ vs Gan^−/−^;TgPer mice, TgPer vs Gan^−/−^;TgPer mice) in the spinal cord. ***E***, Image shows the neuromuscular junctions immunostaining in the gastrocnemius muscles. Colors green and red represent Nefl and α-bungarotoxin. Original magnification 63×. Scale bar, 50 µm. ***F***, Graph shows quantitative analysis of fully, partial, and denervated NMJs. The Gan^−/−^;TgPer mice exhibited a significant decrease in the fully innervated NMJs in comparison with WT and Gan^−/−^; **p = 0.0092 (WT vs Gan^−/−^;TgPer); *p = 0.0460 (Gan^−/−^ vs Gan^−/−^;TgPer). A significant increase in the number of partial NMJs was found in the Gan^−/−^;TgPer mice in comparison with WT mice (*p = 0.0195). The Gan^−/−^;TgPer mice also exhibited a significant increase in the number of denervated NMJs in comparison with WT, Gan^−/−^, and TgPer mice; **p = 0.0023, 0.0068 and *p = 0.0226, respectively. At least six sections per mouse with three mice per group were considered for the quantification. Data indicate ± SEM, one-way ANOVA with Bonferroni's multiple-comparisons *post hoc* test.

We further investigated whether the pathologic changes in the Gan^−/−^;TgPer mice were associated with neurodegeneration. To examine the extent of neuronal loss, we performed Nissl staining of brain and spinal cord samples. The neuronal cell body count was done with the use of the particle analysis plug-in from ImageJ software. The Nissl stain count in the cortex of Gan^−/−^;TgPer mice revealed a reduction of the total neuronal population in comparison with Gan^−/−^ mice and TgPer mice (Fig. 8*A*). Neurons larger than 50 µm^2^ in area were counted for the analysis. There was a significant loss of ∼25% neurons in the cortex of Gan^−/−^;TgPer mice in comparison with Gan^−/−^ and TgPer mice (Fig. 8*B*). Similarly, there was a significant loss of ∼30% neurons larger than 150 µm^2^, which correspond to motor neurons, in the spinal cord of Gan^−/−^;TgPer mice compared with WT mice (Fig. 8*C*,*D*).

We investigated whether spinal neuron degeneration was associated with loss of neuromuscular junctions (NMJs) in gastrocnemius muscles. The axons were stained with anti-Nefl (green) and muscle junctions with α-bungarotoxin (red; Fig. 8*E*). Significant loss of fully innervated NMJs occurred in the Gan^−/−^;TgPer (31%) mice in comparison with WT (85%) and Gan^−/−^ (72%) mice, whereas the partially innervated NMJs were significantly increased in Gan^−/−^;TgPer (50%) mice in comparison with WT (12%) mice. Moreover, the denervated NMJs in muscles of Gan^−/−^;TgPer (18%) mice were also significantly increased in comparison with WT (2%), Gan^−/−^ (4%), and TgPer (7%) mice (Fig. 8*F*). Thus, the combined results from Nissl stain and NMJs count suggest that the loss of spinal neurons is associated with significant muscle denervation in the Gan^−/−^;TgPer mice.

### Enlarged axons in dorsal and ventral roots

**Figure 9.**
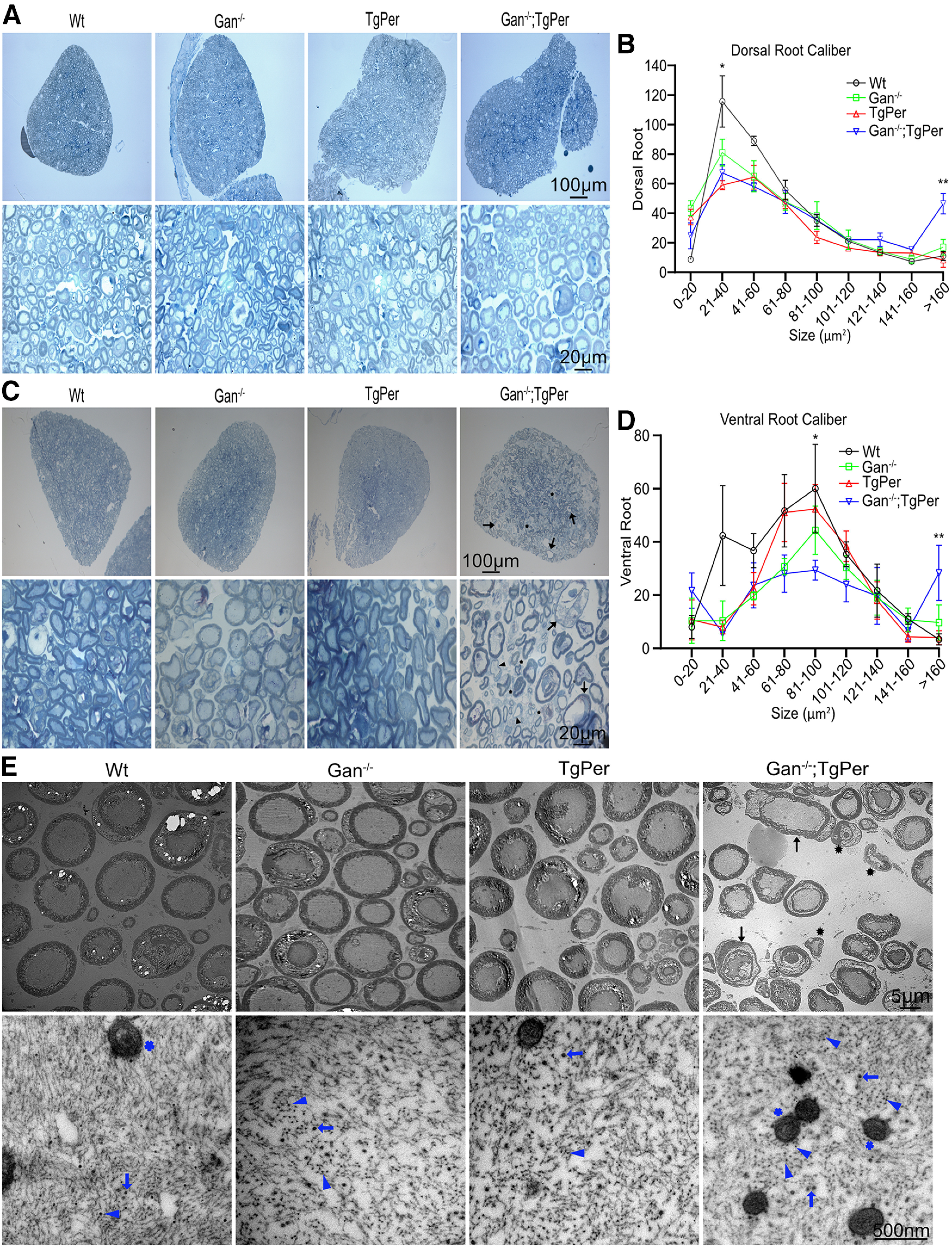
Analyses of dorsal and ventral roots, axonal degeneration and giant axons in Gan^−/−^;TgPer mice. ***A***, ***C***, The toluidine blue staining of dorsal and ventral roots. Original magnification 20× and 100×. Scale bars: 100 µm (upper images ***A*** and ***C***) and 20 µm (lower images ***A*** and ***C***). Three mice were considered in each group. ***B***, ***D***, Graphs represent quantification of dorsal and ventral axon calibers at 12 months of age, respectively. Data represent ± SEM; *p = 0.0193 (WT vs TgPer mice), *p = 0.0424 (WT vs Gan^−/−^;TgPer mice) in dorsal axon caliber 21–40 µm^2^; **p = 0.0062 (WT vs Gan^−/−^;TgPer mice); *p = 0.0176 (Gan^−/−^ vs Gan^−/−^;TgPer mice); **p = 0.0040 (TgPer vs Gan^−/−^;TgPer mice) in dorsal axon caliber >160 µm^2^. Gan^−/−^;TgPer mice exhibited massive loss of axons in the ventral root (***D***), especially in axons with calibers between 81 and 100 µm^2^; *p = 0.0483 (WT vs Gan^−/−^;TgPer). Significant number of axons with calibers >160 µm^2^ were also detected in the ventral root of Gan^−/−^;TgPer mice; *p* = ** 0.0083 (WT vs Gan^−/−^;TgPer); *p = 0.0437 (Gan^−/−^ vs Gan^−/−^;TgPer); **p = 0.0098 (TgPer vs Gan^−/−^;TgPer). Giant axons (arrow) along with demyelinated (arrowhead) and degenerated (asterisk) axons were found in Gan^−/−^;TgPer ventral root (***C***). ***E***, TEM of ventral roots. Original magnification 400× and 10000×. Scale bars: 5 µm for ***E*** upper images and 500 nm for ***E*** lower images. TEM pictures show giant axons (arrows) with degenerating axons (asterisks) in ventral roots of Gan^−/−^;TgPer mice. Higher magnification of ventral roots revealed trapped mitochondria (blue star) surrounded by IF aggregates (blue arrowheads) and microtubules (blue arrows) in Gan^−/−^;TgPer mice.

Light microscopy of semithin sections of dorsal and ventral roots stained with toluidine blue was performed to analyze changes in number and size of axons. The dorsal root did not exhibit major loss of sensory axons in Gan^−/−^;TgPer mice when compared with WT, Gan^−/−^, and TgPer mice (Fig. 9*A*). Nonetheless, there was a significant reduction of axons in size between 20 and 40 µm^2^ in Gan^−/−^;TgPer mice when compared with WT mice (Fig. 9*B*). Moreover, the number of dorsal root axons larger than 160 µm^2^ was significantly increased in Gan^−/−^;TgPer mice when compared with WT, Gan^−/−^, and TgPer mice. As expected from the substantial loss of spinal neurons, microscopy of ventral roots revealed massive loss of axons in Gan^−/−^;TgPer mice (Fig. 9*C*). For instance, there was a significant reduction in ventral root axons with calibers in the range of 81–100 µm^2^ in the Gan^−/−^;TgPer mice (Fig. 9*D*). Moreover, microscopy revealed abnormally high number of axons with calibers larger than 160 µm^2^, including giant axons of 200 µm^2^ and more in size (Fig. 9*C*,*D*, black arrows). The swollen axons (black arrows) in ventral roots in Gan^−/−^;TgPer were often associated with shredded myelin or thin myelin sheet. Electron microscopy confirmed the presence of giant axons with neurofilament accumulations in the Gan^−/−^;TgPer mice at 12 months of age, along with degenerated axons with shredded myelin (Fig. 9*E*). High magnification revealed axons with disorganized NFs sometimes surrounded by mitochondria. In contrast, there was no evidence of such axonal degenerative changes in ventral roots from Gan^−/−^ mice and TgPer mice (Fig. 9*E*).

## Discussion

Here, we report the creation and characterization of a new mouse model for GAN that is based on the overexpression of Prph transgene in the context of targeted disruption of the Gan gene. The Gan^−/−^;TgPer mice were generated by breeding previously described Gan^−/−^ mice (Dequen et al., 2008) with transgenic mice overexpressing peripherin (TgPer; Beaulieu et al., 1999). In contrast to previously reported Gan^−/−^ mice that did not develop significant behavioral and pathologic phenotypes (Valle et al., 2006; Dequen et al., 2008; Ganay et al., 2011), the Gan^−/−^;TgPer mice exhibit cognitive, sensory, and motor deficits reminiscent of GAN disease. The Gan^−/−^;TgPer mice are characterized by the formation of large IF accumulations composed of Prph and type IV NF proteins, which can produce giant axons (≥160 μm^2^) in the dorsal and ventral roots (Fig. 9). The disease in this new mouse model evolves with significant loss of cortical neurons and spinal motor neurons (Fig. 8). It can be concluded from this model that NF disorganization triggered by Prph overexpression in the context of Gan deficiency is sufficient to cause neurodegeneration.

The molecular mechanisms underlying GAN pathogenesis have remained poorly understood, in part because of the lack of a mouse model mimicking the severe phenotypes of the human GAN disease. Gigaxonin is a protein comprising a BTB-Kelch domain and an adaptor of the Cul3-E3 ubiquitin ligase complex. Evidence suggests that the mutations in the GAN gene generally confer a loss of function of gigaxonin. The most recognized substrates of gigaxonin are IF proteins. Thus, cell culture studies demonstrated a role of gigaxonin in the degradation of IF proteins via a proteasomal-dependent pathway (Mahammad et al., 2013). This could explain why GAN is associated with large accumulations of NFs, a hallmark of the disease. However, it has remained unknown to what extent the abnormal NF accumulations in GAN play a causative role in neuronal dysfunction and degeneration. Gigaxonin may also act on other substrates. For instance, gigaxonin-E3 ligase was found to be a regulator of Shh activation by mediating the degradation of the Patched (Ptch) receptor. Suppression of gigaxonin in zebrafish larvae caused Shh signaling impairment and neurodevelopmental deficits (Arribat et al., 2019). Yet, to what extent Shh dysfunction contributes to symptoms in GAN patients remains to be investigated.

The results presented here support the idea that disorganization of neuronal IFs triggered by the suppression of the Gan gene can drive neurologic phenotypes and neurodegenerative changes. As shown in Figure 2*H*, the Gan^−/−^ mice and TgPer transgenic mice exhibited higher levels of Prph and Nefl than WT mice. However, the excess levels of Prph in these mice are not sufficient to cause large IF accumulations and disease phenotypes within 12 months of age. In contrast, overexpression of the Prph transgene in the context of Gan^−/−^ in Gan^−/−^;TgPer mice caused an additional boost in Prph levels that was sufficient to trigger large IF accumulations at 3 months of age (Fig. 2*Eiv*,*Fiv*,*Giv*). This suggests that a threshold level of Prph was required to provoke NF disorganization with ensuing sensory and motor deficits (Fig. 2*A*,*C*).

At 12 months of age, the Gan^−/−^;TgPer mice exhibited symptoms reminiscent of GAN disease including cognitive, sensory, and motor impairment, unlike the single transgenic TgPer mice and Gan^−/−^ mice. The Gan^−/−^;TgPer mice developed memory decline and anxiety symptoms (Fig. 3*B*,*D*). Moreover, the Gan^−/−^;TgPer developed sensory deficits reminiscent of GAN disease (Fig. 3*F*,*G*). Further, the Gan^−/−^;TgPer mice exhibited severe motor impairment reflected by abnormal gait (Fig. 3*K–M*) and poor performance in the hindlimb extension, rotarod, and grid tests (Fig. 3*H–J*). These overt phenotypes were associated with GAN-like pathologic changes (Berg et al., 1972; Bomont et al., 2003; Hentati et al., 2013), such as abnormal neuronal IF accumulations and neuronal loss in the brain and spinal cord (Fig. 8*A–D*). Further, this loss of spinal neurons is associated with muscle denervation (Fig. 8*E*,*F*). The IF inclusion bodies formed in brain cortical neurons of Gan^−/−^;TgPer mice were composed of Prph together with Nefl (Figs. 5, 6*B*). In spinal neurons, the excess Prph levels in Gan^−/−^;TgPer mice caused large NF accumulations in the perikaryon and axon (Figs. 4, 6*A*). The NF disorganization led to the formation of giant axons, a hallmark of the disease (Asbury et al., 1972), in ventral and dorsal roots of Gan^−/−^;TgPer mice. Thus, axons with a caliber larger than 160 µm^2^ were frequent in these mice, and some axons had a caliber of up to 300 µm^2^. With electron microscopy, the ventral root of Gan^−/−^;TgPer revealed disorganized IFs (Fig. 9*E*, blue arrowheads). Along with IF aggregates were trapped mitochondria (Fig. 9*E*, blue star) surrounded by microtubules (Fig. 9*E*, blue arrows). Microscopy of toluidine blue–stained ventral roots revealed a significant loss of motor axons in Gan^−/−^;TgPer (Fig. 9*C*,*D*), in accordance with the loss of spinal neurons (Fig. 8*C*).

The disorganization of neuronal IFs mediated by gigaxonin deficiency may provoke multiple deleterious effects resulting in neuronal dysfunction or neurodegeneration. Previous studies demonstrated that abnormal distribution of NFs can lead to changes in axonal caliber and reduced conductivity (Zhu et al., 1997; Beaulieu et al., 2002). Axonal transport defects have also been reported in some mouse models with NF disorganization (Collard et al., 1995; Millecamps et al., 2007; Perrot and Julien, 2009). Of potential relevance to GAN was the finding that NF proteins are involved in synaptic function. For instance, a depletion of Nefl in mice caused a reduction in levels of glutamate receptor GluN1 with an ensuing decrease of hippocampal dendritic spines and depressed long-term potentiation induction (Yuan et al., 2018). The availability of a new mouse model of GAN with severe cognitive, sensory, and motor phenotypes described here provides a new tool to investigate the pathologic changes associated with gigaxonin deficiency and to examine whether synaptic dysfunction is associated with NF disorganization. Furthermore, the Gan^−/−^;TgPer mice should provide a unique animal model for testing therapeutics.
